# 2036. Dual VIM-producing *Proteus mirabilis*, Including a Novel VIM-75, Among Elderly Patients in a Medical Center from Hungary: Report from the 2020 SENTRY Antimicrobial Surveillance Program

**DOI:** 10.1093/ofid/ofac492.1658

**Published:** 2022-12-15

**Authors:** Lalitagauri M Deshpande, Katalin Burián, Ilona Dóczi Csányi, Mariana Castanheira

**Affiliations:** JMI Laboratories, North Liberty, Iowa; University of Szeged, Szeged, Csongrad, Hungary; Albert Szent-Gyorgyi Medical Centre, Szeged, Csongrad, Hungary; JMI Laboratories, North Liberty, Iowa

## Abstract

**Background:**

Nonsusceptibility of *Proteus mirabilis* to imipenem allows this pathogen evade surveillance for carbapenemases. Metallo-beta-lactamases of the VIM family are widespread among Enterobacterales in Europe. We identified an outbreak of *P. mirabilis* carrying 2 VIM enzymes among elderly patients from a medical center in Hungary.

**Methods:**

A total of 16 *P. mirabilis* isolates were received from Hungary during 2020 as part of the SENTRY Antimicrobial Surveillance Program. Isolates were susceptibility tested by the CLSI reference broth microdilution method. Carbapenem-nonsusceptible isolates were submitted to whole genome sequencing, screened for resistance mechanisms, and evaluated for core genome multi-locus sequence typing (cgMLST).

**Results:**

Among the 16 *P. mirabilis* isolates from Hungary, 5 carbapenem-nonsusceptible, multidrug-resistant isolates (3 from urinary tract infections and 1 each from bloodstream infection and pneumonia) were identified from patients 66-92 years old. Four of these 5 isolates were listed as community acquired. All isolates were resistant to ceftriaxone (MIC, > 8 mg/L), cefepime (16- > 32), imipenem ( > 8), gentamicin ( > 16), levofloxacin (16- > 32), nitrofurantoin ( > 64), trimethoprim/sulfamethoxazole ( > 4), and plazomicin ( > 128), but susceptible to meropenem (0.25-0.5) and ertapenem (0.03-0.25). Isolates carried *bla*_VIM-4_ and *bla*_VIM-75_ on a class 1 integron within *IS26* and separated by *aac(6’)-IIc*. *IS26* was located on a compound plasmid carrying other resistance-encoding genes, including *armA*. The integron structure is identical to the *bla*_VIM-4_ and *bla*_VIM-1_-carrying integron described from a *Vibrio cholerae* isolated from a seagull in France in 2015 (KR262557). *bla*_VIM-75_ is a single amino acid variant (Q60R) of *bla*_VIM-1_. All isolates carried mutations in the QRDR (*gyrA*_S83I, *parC*_S84I). Based on cgMLST analysis, these 5 *P. mirabilis* isolates were highly similar, with only 7-19 SNPs detected.

**Conclusion:**

The outbreak of multidrug-resistant *P. mirabilis* isolates carrying *bla*_VIM-4_ and *bla*_VIM-75_ in a Hungarian medical center is a cause of concern. Surveillance should be performed to understand the spread of and treatment options for infections caused by carbapenem-nonsusceptible *P. mirabilis*.

**Disclosures:**

**Lalitagauri M. Deshpande, PhD**, Melinta: Grant/Research Support|Pfizer: Grant/Research Support **Katalin Burián, MD**, JMI Laboratories: Grant/Research Support **Ilona Dóczi Csányi, MD**, JMI Laboratories: Grant/Research Support **Mariana Castanheira, PhD**, AbbVie: Grant/Research Support|Cidara: Grant/Research Support|GSK: Grant/Research Support|Melinta: Grant/Research Support|Pfizer: Grant/Research Support|Shionogi: Grant/Research Support.

